# Cerebral Palsy – Early Diagnosis and Intervention Trial: protocol for the prospective multicentre CP-EDIT study with focus on diagnosis, prognostic factors, and intervention

**DOI:** 10.1186/s12887-023-04312-7

**Published:** 2023-10-30

**Authors:** Christina Engel Hoei-Hansen, Lene Weber, Mette Johansen, Rebecca Fabricius, Jonas Kjeldbjerg Hansen, Anne-Cathrine F. Viuff, Gitte Rønde, Gitte Holst Hahn, Elsebet Østergaard, Morten Duno, Vibeke Andrée Larsen, Camilla Gøbel Madsen, Katrine Røhder, Ann-Kristin Gunnes Elvrum, Britt Laugesen, Melanie Ganz, Kathrine Skak Madsen, Maria Willerslev-Olsen, Nanette Mol Debes, Jan Christensen, Robin Christensen, Gija Rackauskaite

**Affiliations:** 1 Department of Paediatrics and Adolescent Medicine, University Hospital Rigshospitalet, Blegdamsvej 9, 2100 Copenhagen, Denmark; 2 Department Clinical Medicine, University of Copenhagen, Copenhagen, Denmark; 3 Department of Occupational Therapy and Physiotherapy, Copenhagen University Hospital, Rigshospitalet, Copenhagen, Denmark; 4 Section for Biostatistics and Evidence-Based Research, the Parker Institute, Bispebjerg and Frederiksberg Hospital, Copenhagen, Denmark; 5 Department of Paediatrics and Adolescent Medicine, University Hospital Aalborg, Aalborg, Denmark; 6 Department Clinical Medicine, University of Aarhus, Aarhus, Denmark; 7 Department of Paediatrics and Adolescent Medicine, University Hospital Herlev, Herlev, Denmark; 8 Department of Neonatology, University Hospital Rigshospitalet, Copenhagen, Denmark; 9 Department of Clinical Genetics, University Hospital Rigshospitalet, Copenhagen, Denmark; 10 Department of Radiology, University Hospital Rigshospitalet, Copenhagen, Denmark; 11 Radiological Section, Centre for Functional and Diagnostic Imaging and Research, Copenhagen University Hospital, Hvidovre, Denmark; 12 Department of Psychology, University of Copenhagen, Copenhagen, Denmark; 13 Department of Neuromedicine and Movement Science (INB), Norwegian University of Science and Technology, Norwegian University of Science and Technology, Trondheim, Norway; 14 Clinical Nursing Research Unit, Aalborg University Hospital, Centre for Clinical Guidelines, Aalborg University, Aalborg, Denmark; 15 Datalogisk Institut, Københavns Universitet, Copenhagen, Denmark; 16 Neurobiologisk Forskningsenhed, Rigshospitalet, Copenhagen, Denmark; 17 Danish Research Centre for Magnetic Resonance, Centre for Functional and Diagnostic Imaging and Research, Copenhagen University Hospital - Amager and Hvidovre, Copenhagen, Denmark; 18 Department of Neuroscience, University of Copenhagen and Elsass Foundation, Copenhagen, Denmark; 19 Section of Social Medicine, Department of Public Health, University of Copenhagen, Copenhagen, Denmark; 20 Research Unit of Rheumatology, Department of Clinical Research, University of Southern Denmark, Odense University Hospital, Odense, Denmark; 21 Department of Paediatrics and Adolescent Medicine, Aarhus University Hospital, Aarhus, Denmark

**Keywords:** Cerebral palsy, Early diagnosis, General movements assessment, Genomics, Hand assessment for infants, Brain imaging, Intervention

## Abstract

**Background:**

Early diagnosis of cerebral palsy (CP) is important to enable intervention at a time when neuroplasticity is at its highest. Current mean age at diagnosis is 13 months in Denmark. Recent research has documented that an early-diagnosis set-up can lower diagnostic age in high-risk infants. The aim of the current study is to lower diagnostic age of CP regardless of neonatal risk factors. Additionally, we want to investigate if an early intervention program added to standard care is superior to standard care alone.

**Methods:**

The current multicentre study CP-EDIT (Early Diagnosis and Intervention Trial) with the GO-PLAY intervention included (Goal Oriented ParentaL supported home ActivitY program), aims at testing the feasibility of an early diagnosis set-up and the GO-PLAY early intervention. CP-EDIT is a prospective cohort study, consecutively assessing approximately 500 infants at risk of CP. We will systematically collect data at inclusion (age 3–11 months) and follow a subset of participants (*n* = 300) with CP or at high risk of CP until the age of two years. The GO-PLAY early intervention will be tested in 80 infants with CP or high risk of CP.

Focus is on eight areas related to implementation and perspectives of the families: early cerebral magnetic resonance imaging (MRI), early genetic testing, implementation of the General Movements Assessment method, analysis of the GO-PLAY early intervention, parental perspective of early intervention and early diagnosis, early prediction of CP, and comparative analysis of the Hand Assessment for Infants, Hammersmith Infant Neurological Examination, MRI, and the General Movements method.

**Discussion:**

Early screening for CP is increasingly possible and an interim diagnosis of “high risk of CP” is recommended but not currently used in clinical care in Denmark. Additionally, there is a need to accelerate identification in mild or ambiguous cases to facilitate appropriate therapy early. Most studies on early diagnosis focus on identifying CP in infants below five months corrected age. Little is known about early diagnosis in the 50% of all CP cases that are discernible later in infancy. The current study aims at improving care of patients with CP even before they have an established diagnosis.

**Trial registration:**

ClinicalTrials.gov ID 22013292 (reg. date 31/MAR/2023) for the CP-EDIT cohort and ID 22041835 (reg. date 31/MAR/2023) for the GO-PLAY trial.

## Introduction

Cerebral palsy (CP) is the most common cause of lifelong motor impairment in children and is defined by the Surveillance of Cerebral Palsy in Europe (SCPE) as “a group of permanent but not unchanging disorders of movement and/or posture and of motor function, which are due to a non-progressive interference, lesion, or abnormality of the developing/immature brain” [1]. An early diagnosis is important in order to start relevant intervention, when neuroplasticity is highest [2]. Research suggests that early intervention programs have a positive influence on cognitive and motor outcomes and are a parental request [3].

In 2010 the Danish Cerebral Palsy Follow-up Program (CPOP) was introduced with the aim of establishing more standardized healthcare for children with CP [4]. The CPOP ensures that children with CP are followed annually and aims to document, monitor, and improve the quality of health care for children with CP in Denmark. A previous Danish national study from birth years 1995–2003 found that the median corrected diagnostic age of children with CP was 11 months based on first mention in the patient file [5]. In a recent study, the diagnostic age was 13 months for children with CP born in 2010–2013, based on the first registered diagnosis in The National Patient Registry [6].

The best predictive tools for early diagnosis of CP have been found to be the Prechtl Qualitative Assessment of General Movements (GMA) [7], Hammersmith Infant Neurological Examination (HINE) [8]and cerebral magnetic resonance imaging (MRI) [3]. These tools can be used to diagnose CP before the age of five months [3]. The method Hand Assessment for Infants (HAI) [9] has in recent publications been found promising to complement these tests, but little evidence on HAI has been published. Feasibility of an early-diagnosis set-up of a systematic approach with neurological assessment, GMA/HINE and neuroimaging has been evaluated in several studies [10–12], but not yet in Denmark, and not in a setting where both the newborn- and infant-detectable risk pathways were included. Neonates may have obvious risk factors for CP at birth/in the neonatal period. In the present study, we will also include infants without such risk factors. These children from the infant-detectable risk pathway present with clinical findings in the first year of life suggesting CP.

More than 80% of children with CP have abnormal brain imaging [13]. It has been recommended for many years to use MRI when diagnosing CP, even though CP remains a clinical diagnosis and MRI can be normal in a child with CP [3]. A systematic review from 2007 showed that the majority of MRIs gave clues to the pathogenesis of CP [14]. In a previous Australian study of children with CP, MRI patterns varied depending on parity, gestational age, level of neonatal care, Apgar score, and time to established respiration [15]. Diagnostic MRI of newborns and infants with suspected CP is based on images with the focus on identifying structural brain pathology, such as periventricular leukomalacia, maldevelopment, or grey/white matter damage.

Genetics is known to play an important role in the development of CP. Previous research with genetic testing has shown that genetic aetiology, while often seen in children with no previous gestational or neonatal risk factor, is not limited to this group nor those with other neurodevelopmental comorbidities such as epilepsy or intellectual disability [16]. One study has suggested an association between CP and genes that hinder early brain development and/or predispose to susceptibility to environmental risk factors [17]. Causes for the various CP subtypes, including the most prevalent spastic subtype and the rare ataxic or dyskinetic subtypes, are different. In some cases, the cause is obvious, such as asphyxia during labour, cerebral bleeding, or infection. In many cases the exact cause is unknown. However, in an increasing number of patients it is now possible to identify the underlying cause, since it has become clear that CP can be part of several genetic syndromes. Many treatable metabolic and genetic diseases such as dopa-responsive dystonia may be misdiagnosed as CP [18, 19]. Identifying a genetic cause makes it possible to provide genetic counselling and maybe a specific treatment, e.g., with nutrient-specific diets or medications, which can interrupt disease progression and prevent further injury. In the current study extensive genetic testing with whole genome sequencing will be performed in participants with definite CP or high risk of CP.

Receiving an early diagnosis of CP or high risk of CP is of high priority and parents and care providers agree that early access to interventions is important for the child [20]. Parents of children at risk of CP may experience high stress levels, depression, and chronic sorrow symptoms [21, 22]. Intensive home-based approaches addressing cognitive and motor improvement increasingly involve parents as treatment providers, supervised by therapists [23]. This can lead to both a positive parental outcome such as increased competences and motivation for care of the child, knowledge of the disease, as well as negative outcomes, because parents may be overwhelmed by the burden of responsibility and the feeling of insufficiency or lack of confidence [24].

The current study CP-EDIT (Early Diagnosis and Intervention Trial) with the GO-PLAY intervention included (Goal Oriented ParentaL supported home ActivitY program), aims to test the feasibility of an early diagnosis and intervention set-up in four neuropaediatric centers in Denmark with focus on eight areas related to implementation and the perspective of the families: early cerebral MRI, early genetic testing, implementation of the General Movements Assessment method, analysis of the GO-PLAY early intervention, parental perspective of early intervention, parental perspective of early diagnosis, early prediction of CP, and comparative analysis of the Hand Assessment for Infants method, Hammersmith Infant Neurological Examination, MRI, and the General Movements method.

## Study design and methods

Study protocol is version CP-EDIT_290323 The study is funded by the Elsass Foundation (grant no. 21-B01-1192 and 22-B01-0664), The Research Fund of Rigshospitalet (grant no RH-E-2251–03), The Association of Danish Physiotherapists Research Fund, Region Nordjyllands Sundhedsvidenskabelige Forskningsfond, and the Dronning Louise foundation. The study funders have no influence on the study.

All co-authors of this protocol paper are trial contributors. The steering group will function as data monitoring committee and includes CHH (principal investigator), GR, LW, RF, MJ. Inclusion of participants and placement into the groups in the CP-EDIT prospective cohort (definite CP, high risk of CP, unclear, or definitely not CP) is done in two local steering groups (Eastern Denmark: CHH, LW, NMD, RF; Western Denmark: GR, JH, MJ, RF). One person from the steering group is responsible for each of the participating centres. The randomization for the GO-PLAY study is based on a prepared random assignment number list kept by the statistical advisor (RC), who is not involved in the recruitment. The children will be allocated to groups based on stratified permuted block randomization by age at the first visit (below or more than 6 months) and the HINE score (≤ 40 or > 40) to control the balance for prediction of gross motor development. Eligible participants will be randomly assigned in permuted blocks of 2 and 6, according to computer-generated random numbers, to either GO-PLAY or standard care (SAS Proc Plan). We anticipate that allocation concealment will be successful in preventing selection bias since the statistician conceals the allocation sequence from those assigning participants to the intervention groups, until the moment of assignment; from which point the individual is part of the intention-to-treat (ITT) population. Neither the families nor the therapists responsible for the first data collection are blinded to group allocation. The therapists responsible for data collection at follow-up assessments are not involved in the intervention, blinded to the group allocation, and have no access to previous assessments.

### Availability of data and material

By contacting the corresponding author and upon reasonable request, de-identified data and standard operating procedures can be made available for the majority of the data if there can be established a data transfer agreement. The handling of data was approved by the local Data Protection Agency (j.nr.: P-2022-980). Protocol changes will be communicated to the research ethics committee and trial registry.

The study will be open for participant inclusion April 1^st^, 2023.

#### Dissemination

Findings regarding the CP-EDIT results will be communicated in line with a dissemination plan that will be develop by the steering committee, relevant stakeholders, decision makers, academia, to the public through dissemination activities including publications in peer-reviewed, international medical journals and to all included participants through the laymans summary.

#### Inclusion procedure and follow-up

A three-step procedure will be applied in order to identify as many infants with high risk of CP as possible before the age of 1 year.

##### The 1^st^ step

Newborns- and infants will be screened for eligibility in neonatal departments or when referred to the neuropaediatric clinic in one of the participating hospitals. Referral to the study can also be from parents or from other Danish paediatric departments. Inclusion criteria are listed in Table 1. Four Danish paediatric departments will participate (Copenhagen University Hospital—Rigshospitalet, Copenhagen University Hospital—Herlev, Aarhus University Hospital, and Aalborg University Hospital). Inclusion period is April 1st, 2023, to March 31st, 2025.

**Table 1:** Inclusion criteria CP-EDIT study

Newborn-detectable pathway	Infant detectable pathway
1. Preterm birth with gestational age below 32 or birth weight < 1500 g and clinical concern 2. Moderate to severe brain injury (cystic periventricular leukomalacia, Papile grade 3 to 4 intraventricular haemorrhage, neonatal stroke, term hypoxic-ischaemic encephalopathy (≥ 35 weeks gestation at birth) or other significant neurological condition) 3. History with known risk factors for CP (e.g. neonatal seizures, meningitis, kernicterus, severe hypoglycaemia, brain malformation, increased tone, ExtraCorporal Membrane Oxygenation) 4. Parental concern and one of the factors above	1. Inability to sit independently by age 9 months 2. Hand function asymmetry or crawl asymmetry 3. Inability to take weight through the plantar surface of the feet 4. History with known risk factors for CP as in neonatal pathway 5. Parental concern and one of the factors above

Inclusion criteria are listed in Table 1.

Exclusion criteria are:

1. Progressive or neurodegenerative disorders.
2. Known genetic or disability disorders not associated with CP, for example Down syndrome.

**Fig. 1:**
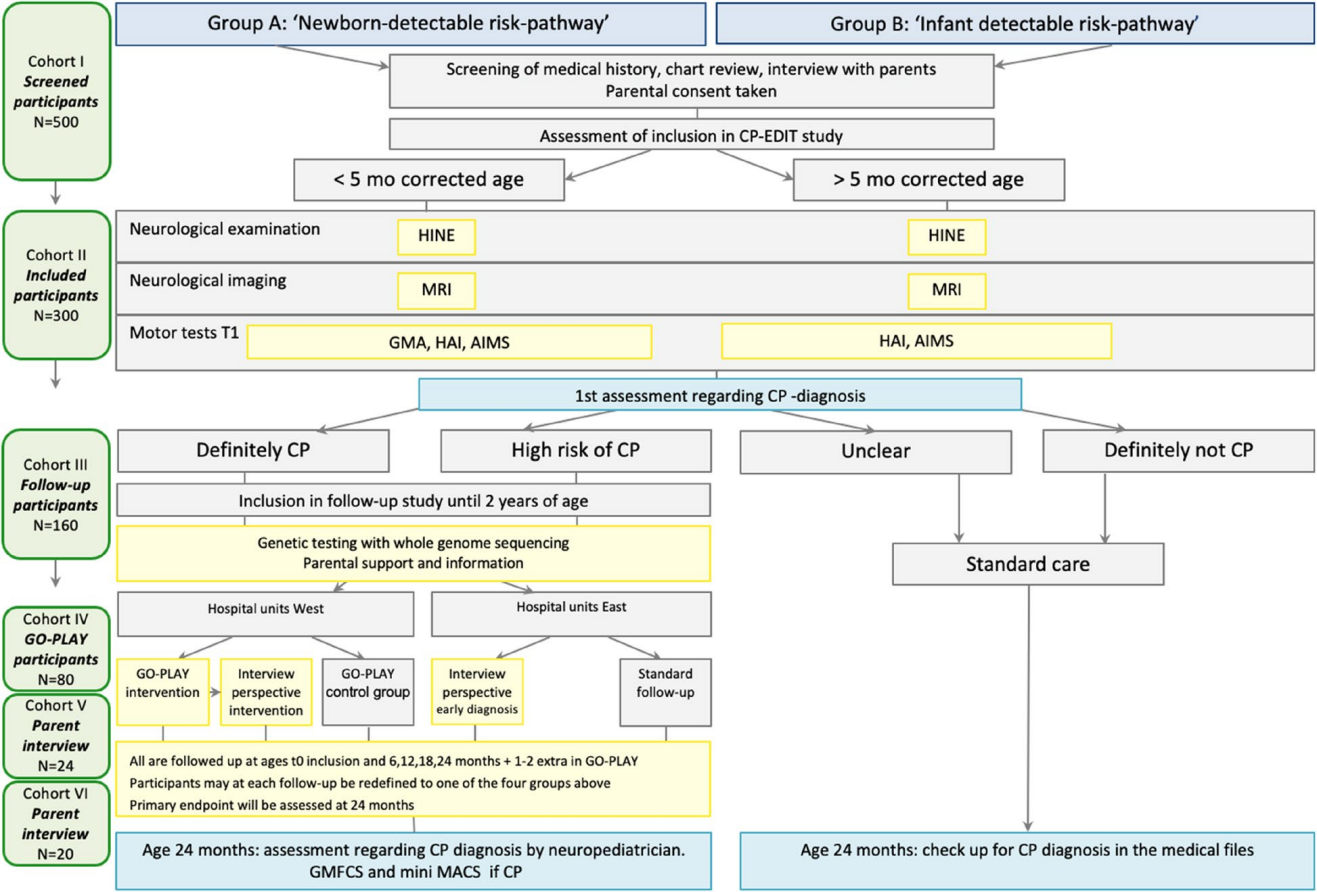
Flow-chart of the CP-EDIT study. Abbreviations: AIMS = Alberta infant motor scale; CP = cerebral palsy; GMA = general movements assessment; GMFCS = gross motor function classification system; HAI = hand assessment for infants; HINE = Hammersmith infant neurological examination; MACS = manual ability classification system; MRI = magnetic resonance imaging; n = number

Flow chart of the study is depicted in Fig. 1. If one of the inclusion criteria is present and no exclusion criteria is present at the time of screening, a member of local steering committee will be contacted by phone or mail. These children will define cohort I. We expect to screen approximately 500 children in this 1^st^ step.

##### The 2^nd^ step

A child will be invited for the first assessment if the members of local steering group consider that the child has an increased risk of CP and parents’ consent is present. Children, participating in the assessment, will define Cohort II. Expected number of assessed children is 300.

##### The 3^rd^ step

Participants from cohort II will be included in the follow-up study (cohort III who have high risk of CP or definitely CP) if they fulfil at least one of the requirements A or B:

A. any two of the following:
  A.1 Neuroimaging predictive of a motor disability including the involvement of one or more of the following structures: sensori-motor cortex, basal ganglia, posterior limb of the internal capsule, pyramidal tracts.
  A.2 GMA test with absent fidgety GMs at fidgety age
  A.3 HINE scores < 57 at 3months or < 60 at 6months or < 63 at 9 months or < 66 at 12 months
B. both of the following:
  B.1 Unilateral brain injury on neuroimaging (MRI or ultrasound) predictive of CP
  B.2 Clinical signs of asymmetry

The parents will be informed that a child has CP or has a high risk of CP. Approx. 160 children are expected to be included in the follow-up as high risk of CP or CP after the 1^st^ assessment.

Exclusion criteria from the 1^st^ step will be applied during the whole study. For example, if a child shows signs of progressive disorder at the age of 12 months and diagnosis becomes clear before the age of 18 months, the child will be moved from the CP-cohort (cohort III) to the non-CP group and not invited for further assessments in the CP-EDIT; the child will be classified as “no CP” at the age of 24 months.

Participants from cohort III from the two study sites at Aarhus and Aalborg hospitals will be included in the GO-PLAY randomized-controlled trial for children with CP or high risk of CP. Participation requires that parents talk sufficient Danish. We expect 60–80 participants in GO-PLAY.

After screening the definition of ‘Definitely CP’ encompasses participants that fulfil SCPE CP clinical criteria and guided by fulfilling the following: 4/5 for children < 5 months, and 3/4 for children ≥ 5 months of the following at screening.

- Delayed motor development without signs of neuromuscular disease (floppy infant, absent reflexes)
- GMA test with absent fidgety GMs at fidgety age
- HINE scores < 57 at 3months or < 60 at 6months or < 63 at 9 months or < 66 at 12 months
- MRI or ultrasound of brain with a lesion in the abovementioned specific areas
- Focal neurological symptoms (hyperreflexia, clonus, dystonia, ataxia, intention tremor) or clinical signs of asymmetry

### Sample size estimation

Sample size and statistical power has not been possible to estimate, since the variance of the outcomes is not known for many of the gathered data. Screening of 500 patients is realistic based on judgement by clinicians in the involved departments (e.g., by amount of neonatal admissions and referrals to neuropediatric clinic) and recruitment to previous studies in Denmark. Number of patients with CP is estimated from the Danish CP registry, indicating an incidence of 20 new patients from each of the 4 participating centers in the last years, therefore 160 is realistic to include in follow-up study, as children with high risk of CP also can be included, and a high interest from families to participate is anticipated. Distribution of participants in cohort II is estimated to derive from the neonatal pathway in 2/3 and from the infant pathway in 1/3 of cases.

The sample size for the GO-PLAY intervention trial has been calculated to be able to detect a large clinical effect (Cohen’s *d* = 0.8) corresponding to a difference between groups for PDMS-2 of 8 units, with greater than 80% statistical power at a 2-sided level of significance of 5%, comparing GO-PLAY (+ Standard of Care) with standard of care. Anticipating a standard deviation of 10.0, 26 patients are required in each of the two groups of primary interest (GO-PLAY versus standard of care). Accounting for 15% dropout rate, an estimated sample size is *n* = 60; approximately 30 participants per group.

Strategies for achieving adequate participant enrolment to reach target sample size includes dissemination to neuropaediatricians, and neonatologists, advertising on websites, and encouragement to disseminate knowledge about the study at paediatric departments in Denmark.

#### Assessments

For overview of schedule of enrolment, interventions, and assessments see Table 2.

**Table 2:** Flow diagram according to SPIRIT recommendations (Standard Protocol Items: Recommendations for Interventional Trials)

**Variable / characteristic**	**Screening**	**Enrolment visit**	**Allocation to cohort III and randomi-zation to GO-PLAY**	**6 months CA**	**12 months CA**	**18 months CA**	**24 months CA**	**Additional visit if** **GO-PLAY** **t = 0**	**Additional visit if** **GO-PLAY** **t = 6**
*Infant characteristic*									
Eligibility screening	x								
Allocation and randomization			x						
Length, weight, head circumference		x		x	x	x	x	x	x
Gestational age		x							
Apgar score		x							
Birth measurements		x							
*CP risk factors*									
Familial disposition CP		x							
Imaging with CT / MRI^a^		x							
Co-morbidities		x		x	x	x	x	x	x
Medication		x		x	x	x	x	x	x
Genomic testing		x							
*Outcome measures*									
HINE [8]		x			x	x	x	x	x
GMA (if below 5 months) at age 12–15 and 16 weeks [7]		x							
PDMS-2 [25]					x	x	x	x	x
AIMS [26]		x		x	x			x	x
HAI (if below 12 months) [9]^b^		x		x	x			x	x
GMFM-66 [27]							x	x	x
BSID-IV-cog [28]					x	x	x		
COPM by interview in intervention group [29]								x	x
PSS questionnaire [30]		x			x		x	x	x
DASS-21 questionnaire [31]		x			x		x	x	x
MPOC-SP&20 questionnaire [27]^c^							x		x
ASQ questionnaire [32]							x		
Clinical assessment of CP diagnosis		x		x	x	x	x		
GMFCS if CP				x	x	x	x		
Mini-MACS if CP							x		
*Intervention*									
GO-PLAY intervention								x	x

*Abbreviations:* *AIMS* Alberta infant motor scale, *ASQ* ages and stages, *BSID-IV, cog* Bayley scale of infant and toddler development III, cognitive part, *CA* corrected age, *CP* cerebral palsy, *COPM* Canadian occupational performance measure, by interview, *GO-PLAY* Goal Oriented ParentaL supported home ActivitY program, *GMA* general movements assessment, *DASS-21* depression, anxiety, and stress scale-21, *GMFCS* gross motor function classification system, *GMFM-66* gross motor function measure, *GMFCS* gross motor function classification system, *HAI* hand assessment for infants, *HINE* Hammersmith infant neurological examination, *MACS* manual ability classification system, *MPOC-SP&20* measures of processes of care – service providers, *MRI* magnetic resonance imaging, *PDMS-2* Peabody developmental motor scale, *PSS* parental stress scale, *t* time point

^a ^Optional repeat MRI at 12 and 24 months. ^b^At enrolment screening-HAI will be used. At subsequent visits the full HAI assessment will be used. ^c^ If included in the GO-PLAY study

##### Cohort I

The participants will be screened based on medical history, chart review and interview with parents regarding whether they should be included in the CP-EDIT study cohort II.

##### Cohort II

Participants in this cohort will be assessed with the following: a) Clinical examination by a neuropaediatrician including neurological assessment with HINE [8]; b) Cerebral MRI, preferably without anaesthesia. The MRI scan may be optional if pre-existing CT or ultrasound has established the aetiology of CP; c) Motor tests consisting for children below five months of age of GMA [7], HAI [9] and Alberta infant motor scale (AIMS [26]) and for children from 5–12 months of HAI and AIMS.

##### Cohort III

Children with definite CP or high risk of CP. All participants are followed at ages 6, 12, 18 and 24 months and blood samples for whole genome sequencing is categorised as described below.

### Objectives

In the CP-EDIT cohort and in the GO-PLAY RCT a large dataset will be gathered. The focus is on eight areas related to implementation and perspectives of the families, and dissemination of data will be distributed in scientific publications according to the following areas:

- *MRI.* To evaluate MRI findings on early diagnostic MRI of participants in cohort II, who later will be categorized as either definite CP, high risk of CP, unclear or definitely not CP.
- *Genetics.* To evaluate genetic findings in early whole genome sequencing of participants in cohort III, which have definite CP or high risk of CP.
- *GMA implementation.* To assess the feasibility of applying The Prechtl Qualitative Assessment of General Movements in a multi-center Danish hospital setting.
- *Prediction of CP*. To determine the clinical utility of the MRI, HINE, HAI, and GMA to predict a confirmed diagnosis of CP at 24 months, in infants referred below 12 months of age.
- *GMA/HINE/MRI vs. HAI*. To compare diagnostic accuracy of sHAI and GMA/HINE/MRI*.*
- *GO-PLAY.* To analyse the effect of the GO-PLAY intervention with early family-centred set-up for children with definite or high risk of CP. For 6 months these participants will receive regular follow-up in the home of the family with physiotherapist and occupational therapist will monitor and strengthen goal-based training.
- *Parents perspective on intervention.* To explore parents’ perspectives on barriers and facilitators involved in early intervention.
- *Parents perspective on early diagnosis.* To analyse interviews of parents’ perspectives of gains and concerns when having an early diagnosis of high risk of CP and how it affects parent-infant interaction.

#### Methods

- *MRI*. Prospective cohort study. Descriptive analysis of MRI findings in the participants related to allocated group and outcome CP at age 24 months.
- *Genetics*. Prospective cohort study. Descriptive analysis of genetic findings in the participants related to allocated group and outcome CP at age 24 months.
- *GMA implementation.* Process evaluation of GMA implementation. Focus on fidelity, acceptability, and contextual factors influencing the feasibility of GMA.
- *Prediction of CP*. Prospective cohort study developing a transparent multivariable prediction model for individual diagnosis and prognosis of CP. We hypothesize that the MRI, HINE and GMA will have predictive validity equivalent to guideline recommendations and that the HAI score will be an important additive predictor to support early diagnosis of unilateral CP.
- Diagnostic test accuracy study. The aim is to determine the diagnostic accuracy (of sHAI for CP detection by using the interim diagnosis based on the combined assessment with MRI, GMA and/or HINE as a composite reference standard. The accuracy of sHAI will be assessed by measures of the test’s ability to detect the presence of high risk of CP.
- *GO-PLAY*. The primary aim of this trial is to compare the effectiveness of the GO-PLAY intervention, relative to the present standard of care, on the early motor development after 6 months in children with a definite or possible diagnosis of CP. The secondary aim is to compare the effectiveness of the GO-PLAY intervention, relative to the present standard of care, on parents’ ability to manage stress and anxiety and perceptions of health care services.
- *Parents perspectives on intervention*. Qualitative study based on focus groups of parents’ perspectives on barriers and facilitators in early intervention 1–3 months after participating in an early intervention program
- *Parents perspectives on early diagnosis*. Qualitative study based on individual semi-structured interviews of parents of infants with a diagnosis of high risk of CP. Parents are selected by purposive sampling and interviews are conducted in the home of the family 2–4 months after diagnosis of high risk of CP.

#### Primary and secondary outcome measures

- *MRI.* Primary results are a description of MRI findings in children included in the CP-EDIT cohort II. The type of MRI findings will be analysed and related to history, presence and type of CP and age of the child. Secondary results are to analyse if cranial ultrasound and/or CT is correlated to MRI findings.
- *Genetics.* A complete overview of pathogenic/likely pathogenic variants identified in the CP-EDIT cohort III and thus providing an overview of the genetic findings in an unselected cohort of children with suspected CP in a clinical setting.
- *GMA implementation.* Fidelity outcomes: Multifaceted training packages provided: (a) number, (b) content, (c) physiotherapists´ perspectives on the utility of sessions. Proportion of high-risk infants screened with GMA. Interrater reliability between the GMA assessors. Acceptability outcomes: Number of GMA videos (a) sent by parents, (b) made by physiotherapists. Additionally, assessment of physiotherapists´ confidence in providing a GMA score. The contextual factors change during the implementation process, these will be continually examined through observations and if relevant interview with stakeholders.
- *Prediction of CP*. The primary outcome is a confirmed diagnosis of CP “yes vs. no” at age 24 months. For infants defined as definite CP or high risk of CP, diagnosis will be confirmed by a neuropaediatrician according to the SCPE criteria [33]. Key secondary outcome: Motor function will be classified according to the Gross Motor Function Classification System (GMFCS), categorized into five levels from walking without limitation (level I) to non-ambulatory function (levels IV and V) [34]. For infants defined as unclear or definitely not CP, medical records will be reviewed for diagnosis and walking ability at the age of 24 months.
- *Diagnostic test accuracy study*. Infants at risk of CP will be assessed with sHAI, a 6-item structured video recorded play session of 5 min of duration. The test is performed in the clinic and assessed by a certified HAI assessor. A combination of MRI, GMA and/or HINE will be applied as the gold standard i.e., the high risk of CP or CP diagnosis reference standard.
- *GO-PLAY*. Patients referred to CP-EDIT trial will be randomized to receive either GO-PLAY intervention or standard care plus assessments. The primary outcome is improvement in motor development evaluated by Peabody Developmental Motor Scale 2 (PDMS-2) from baseline (T0) to the end of intervention (endpoint T6, 6 months post baseline. Key secondary outcomes will be HAI when the child is ≤ 12 months at the end of intervention, AIMS, Gross Motor Function Measure (GMFM-66), Bayley Scale of Infant and Toddler development 4, cognitive part (BSID-4, cog) and parental well-being. Neither the families nor the therapists responsible for intervention are blinded to group allocation. All outcome assessors will be masked to group allocation. The therapists responsible for data collection at each assessment point are not involved in the intervention, are unbiased as to group allocation, and have no access to previous assessments.
- *Parents perspectives on intervention.* Data are analysed using thematic analysis. Results from this study may support clinicians in understanding the parent’s perspectives and customize early home-based intervention to address parents’ expectations, concerns and needs in the Danish health care system.
- *Parents perspectives on early diagnosis.* Data are analyzed using thematic analysis approach, supported by investigator triangulation [35]. Findings are expected to be presented as themes and subthemes. This study may help clinicians to support the parent-infant interaction and tailor counselling to address parents’ experiences, concerns, and needs.

#### Examination and intervention modalities

##### MRI

All included participants in cohort II with risk of CP (*n* = 300) will be offered MRI as quickly as possible or at term if premature. We expect that at least half of the children will have an existing diagnostic MRI scan or have been offered an MRI scan due to clinical indications. The rest of the included children will be referred to a diagnostic MRI scanning at 3 Tesla by the paediatricians in the CP-EDIT program. If the child is not able to cooperate for the duration of the scan, standard sedative, or general anaesthesia in accordance with regular MRI scan protocols of children will be used.

MRI scans will be described clinically by radiologists at the participating hospitals. The findings will be categorised according to SCPE criteria (Surveillance of Cerebral Palsy in Europe). In participants where ultrasound or CT scanning of the patients have already been performed as part of clinical follow-up, the results will be gathered, and additional MRI may be optional. Repeat MRI at ages 12 and 24 months will be optional for participants in cohort III as part of the complementary NIBS-CP project (NeuroImaging of Babies during natural Sleep to assess typical development and Cerebral Palsy), which may provide important biological information about myelination, microstructure, and connectivity of the white matter fibre tracts, as well as the metabolic profile, including markers of neuronal integrity and glial markers, of the brain tissue [36, 37].

##### Genetics

Blood samples will be obtained from participants with definite or high risk of CP (*n* = 160) upon inclusion in CP-EDIT cohort III after informed, written consent has been obtained by the parents. The parents will also be asked to provide a blood sample and written consent for genome sequencing (trio-analysis) to facilitate identification of de novo variants. Sequencing will be performed by the Danish Genome Center as part of their diagnostic data production for children with rare neurological diseases. A clinical geneticist and clinical laboratory geneticist will perform the data analysis and result interpretation. In cases of variants with uncertain significance a board may be consulted for interpretation of significance. The data will be analysed for copy number variation and structural variants as well as sequence variation in known disease genes and mitochondrial DNA. Results will be categorised as either: I) Pathogenic CP-explaining variant, II) Likely pathogenic CP-explaining variant, III) Variant of uncertain significance, IV) Likely benign variant, V) Benign variants (according to the ACMG guidelines) and VI) Pathogenic variant, non-CP disease. Only data from the proband will undergo a full analysis. All analysis will be performed in a diagnostic setting. De novo variants in gene with no known clinical association may be submitted to GeneMatcher (reference PMID: 26220891).

##### Neurological / motor assessments

The following tests and assessments will be performed by experienced and trained physiotherapists, occupational therapists, and paediatricians at the four study sites. Inter-site training and alignment will be ensured by the steering committee.

The central tests used in the study are:

- Hammersmith Infant Neurological Examination (HINE) is a standardized neurological examination for infants aged 3–24 months. It includes three sections: 1) Neurological Exam – tone and movements, 2) Development of Motor Function – head control, sitting, walking, crawling, rolling, and grasping, and 3) State of Behaviour – consciousness, social orientation, and emotional state. The HINE global score ranges from a minimum of 0 to a maximum score of 78 [38]. A score < 57 at 3 months and < 73 at 6 and 12 months indicates high risk of CP, and < 40 at all ages indicates abnormal outcome, usually CP [8]. The HINE total score and asymmetry-score, also provides insight into CP topography (unilateral vs bilateral) [39] and CP motor severity (ambulant vs non-ambulant, GMFCS I–III vs IV–V) [8, 40].
- Hand Assessment for Infants (HAI) is developed for infants aged 3–12 mo. at risk of CP. The test procedure comprises a semi-structured video-recorded play session lasting 10– 15 min (5 min for the screening-HAI), with a wide range of unilateral and bilateral hand movements. The HAI measures the degree and quality of goal-directed actions performed with each hand separately as well as both hands together. It provides a separate score for each hand, illustrating possible asymmetric hand use as well as a criterion referenced measure of general upper limb ability [9].
- General Movement Assessment (GMA) is an observation that evaluates the quality of an infant’s early spontaneous movement patterns. GMA is categorized in writhing movements (from preterm until 6–9 weeks post term age) and fidgety movements (from 9 to 20 weeks post term age), and especially the absence of fidgety movements is highly predictive for later neurological impairments, particularly for CP [41]. An analysis will also be performed of the Motor Optimality Score-Revised. The MOS-R comprises five subcategories: (1) Quality of fidgety; (2) Observed movement patterns; (3) age-adequate movements (4) Observed postural patterns and (5) movement character. The MOS score ranges from a minimum of 5 to a maximum score of 28. An MOS ranging from 25 to 28 is considered optimal; scores from 20 to 24 are mildly reduced and an MOS below 20 requires intervention. A score below 9 indicates a very high risk for neurodevelopmental disabilities, especially for non-ambulatory CP (MOS-R).

##### The GO-PLAY intervention

**Fig. 2:**
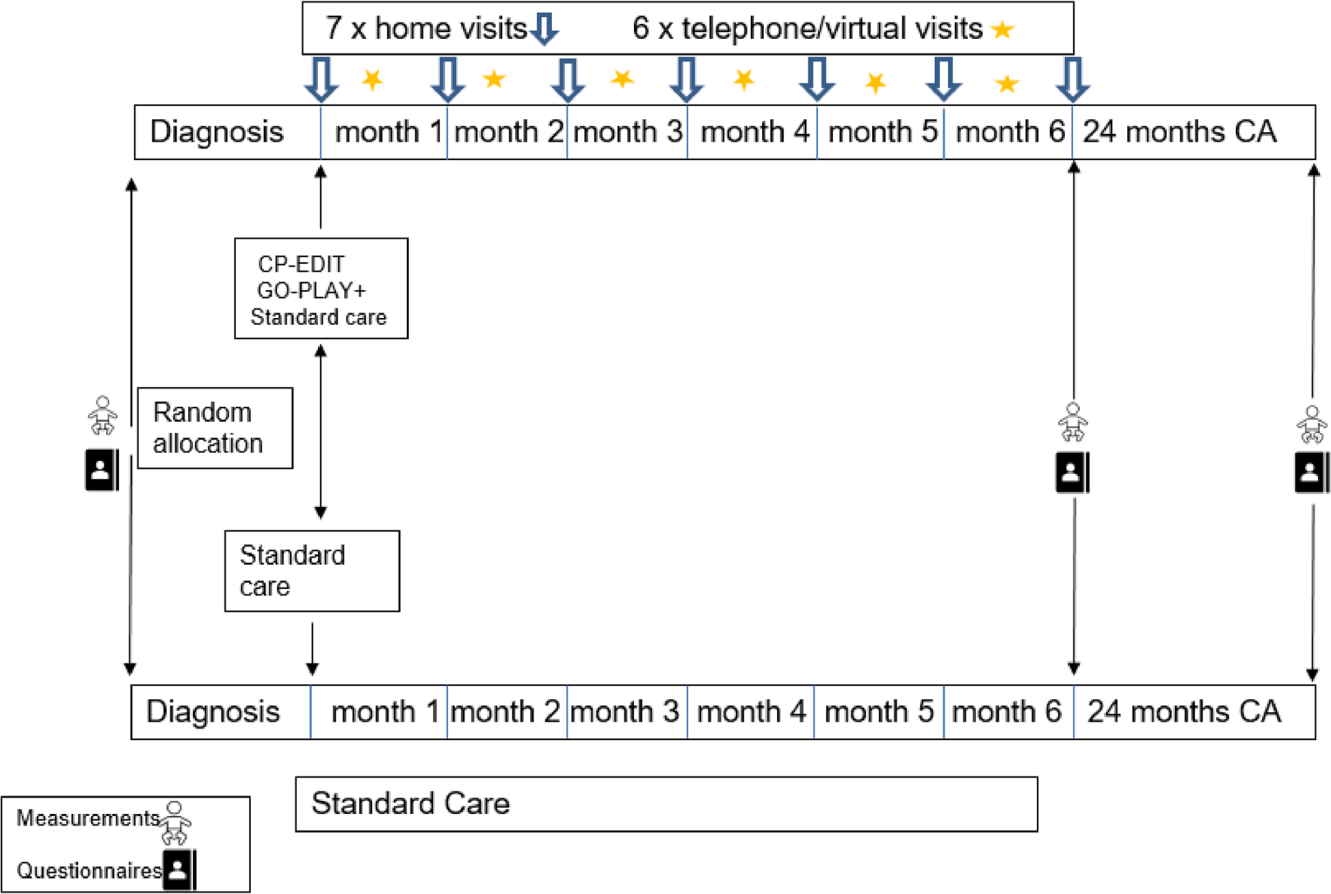
The GO-PLAY intervention. Abbreviations: CA = corrected age; CP-EDIT = cerebral palsy – early diagnosis and intervention trial; GO-PLAY = Goal Oriented ParentaL supported home ActivitY program

The GO-PLAY intervention will last for six months (around 24 weeks) and consist of a home visit once a month (60–90 min) and a virtual meeting (30–45 min minutes) by telephone or video once a month (see Fig. 2). The intervention will be family centred and follow recommendations from international guidelines [41]. An experienced physiotherapist and occupational therapist will be the primary therapists providing the intervention to ensure uniformity. The motor learning element of the intervention is based on the principles of motor learning and dynamic systems theory. Emphasis is on children’s self-initiated actions, which are stimulated by meaningful and motivating activities and toys. The child’s play preferences to elicit self-generated motor activity will be the underlying basis of the training. Minimal manual guidance is provided when needed and withdrawn when the child demonstrates self-generated ability to complete part of the task.

## Discussion and dissemination

Early screening for CP is increasingly possible and an interim diagnosis of “high risk of CP” is recommended to accelerate identification in mild or ambiguous cases and to facilitate appropriate therapy at an age where the infant brain has the highest neuroplastic potential, while further diagnostic assessment occurs [3]. Diagnosing CP can be difficult because infant motor skills are developing, and the presence and absence of hypertonia changes and evolves.

Half of the infants with CP have high risk indicators in the newborn period (e.g., prematurity and encephalopathy). Almost all studies on early diagnosis focus on identifying CP in infants below five months corrected age, because these infants are more often seen in follow-up programs [3]. In this population there is a strong recommendation based on high quality evidence for the use of HINE, GMA, MRI, which in the neonatal population have predictive values around 90% [39, 42, 43]. Little has been published about early diagnosis in the 50% of all CP cases that are discernible later in infancy. These infants may have uneventful pregnancy and birth, and the first notice of CP may be delayed motor milestones or asymmetry in hand function. Often a unilateral CP becomes evident later [3]. For infants older than five months there is also a strong recommendation for HINE test and conditional recommendations for MRI and supplemental motor assessments to improve triangulations of findings [3].

GMA is currently considered central in the assessment before five months of age in recent guidelines. A previous study has shown that the HAI demonstrates overall accuracy ranging from very good to excellent in predicting unilateral CP in infants with high risk of CP between 3.5–12 months of age [44]. The HAI can play a role in diagnosing unilateral CP at an early age in infants born at term as well as infants born preterm [45]. By combining the HAI with neonatal MRI, gestational age, and sex it is possible to accurately identify the prognostic risk of unilateral CP as early as 3.5 to 4.5 months in infants with asymmetric perinatal brain injury [44].

Recent international guidelines suggest early therapy interventions that promote infant neuroplasticity and emphasize family-centred interventions based on the principles of motor-learning (task-specific), enriched environment, parental coaching, and relatively high dosing [41]. Home training is a useful strategy to increase the dose of therapy [46] and it allows the children to practice activities within the context of their everyday lives [3, 41]. A recent study on early intervention for infants with high risk of CP based on the active participation of the family and environmental enrichment (GAME) resulted in advanced motor and cognitive outcomes when compared with standard care [47]. The study involved infants aged between 3–6 months corrected age (CA). Another study (Small Step) included infants at 4–9 months of corrected age and provided early intervention targeted hand use, mobility, and communication during specific periods. They found no group effect for their motor outcome at the end of treatment compared with standard care. Motor development was associated with baseline assessments in the standard care group, while infants in the intervention group developed independent of the baseline level, implying that the intervention helped the most affected children to catch up by the end of treatment, which was sustained at 2 years of age [48].

Parents of children with CP are more prone to stress and anxiety [49]. Parent involvement in early intervention may improve cognitive and motor outcomes in the infant [41] as well as parental outcomes such as improved confidence, motivation, self-efficacy, and increased adherence [50].

The four hospitals in this study represent the two largest neuropaediatric centres in Denmark and two large regional hospitals. Together we have a unique opportunity to investigate early detection of CP and to implement the new tools for early diagnosis. The methods used in the study are well-established at the study centers except GMA, HINE, and HAI, where implementation hands-on by therapists/paediatricians in the involved departments with educational courses is integrated in the study.

The study CP-EDIT aims by the described multiple focus areas to improve early diagnosis of CP and to implement an early intervention program.

## Data Availability

All data generated or analysed during this study will be included in the planned published papers and the supplementary information files.

## Abbreviations

AIMS: Alberta infant motor scale
CP: Cerebral palsy
ASQ: Ages and stages
BSID-III, cog: Bayley scale of infant and toddler development III, cognitive part
CP-EDIT: Cerebral Palsy-Early Diagnosis and Intervention Trial
COPM: Canadian occupational performance measure, by interview
CPOP: Cerebral palsy follow-up program
GMA: Prechtl Qualitative Assessment of General Movements
DASS-21: Depression, anxiety, and stress scale-21
GMFCS: Gross motor function classification system
GMFM-66: Gross motor function measure
GO-PLAY: Goal Oriented ParentaL supported home ActivitY program
HAI: Hand Assessment for Infants
HINE: Hammersmith Infant Neurological Examination
MACS: Manual ability classification system
MPOC-20: Measures of processes of care
MRI: Magnetic resonance imaging
PSS: Parental stress scale
SCPE: Surveillance of Cerebral Palsy in Europe
